# Editorial: Emerging diseases and diagnostics in poultry production

**DOI:** 10.3389/fvets.2025.1664602

**Published:** 2025-08-21

**Authors:** Cuiteng Chen, Chen Zhen, Yu Huang, Chunhe Wan, Chunhua Zhu

**Affiliations:** ^1^Institute of Animal Husbandry and Veterinary Medicine, Fujian Academy of Agricultural Sciences, Fuzhou, China; ^2^Fujian Key Laboratory for Avian Diseases Control and Prevention, Fujian Academy of Agricultural Sciences, Fuzhou, China

**Keywords:** avian diseases, emerging and re-emerging, variant, detection, differentiation, diagnostic

In the realm of avian production, a critical component of global food security and economic stability, the risk and impact of disease challenge are significantly heightened due to large, close confinement rearing systems. These systems, while designed to maximize productivity and improve economies of scale, inadvertently increase the probability of infectious disease spread and the emergence of new poultry disease variants. This has led to substantial economic losses in the poultry industries worldwide, including chickens, ducks, geese, pigeons, and other birds, particularly in China. It's critical challenge to manage the risk and consequence of disease control, due to the economical goal of poultry industry (1).

The primary aim of this Research Topic is to collate works from researchers specializing in virology, bacteriology, disease transmission and mechanisms, and diagnostics. The objective is 3-fold: to understand the current, emerged, re-emerged, and emerging diseases within the poultry industry; to explore diagnostic technologies that could potentially contribute to the development of new diagnostic tools; and to employ new strategies to eradicate potential pathogenic threats to the poultry industry (2).

The emergence and re-emergence of avian diseases, especially for the novel variant diseases, are challenging to control due to lack of effective treatment options, that pose the biggest threat to the poultry industry. For disease control purposes, it's the key point to make an accurate diagnosis and collect information with variables relevant to flock health, including prevalence or incidence of infection, morbidity and mortality rates, flock immunity and distribution of antibody titers, production records, etc.

Fan et al. developed a novel triplex fluorescence reverse transcription-loop-mediated isothermal amplification (TLAMP) assay in which traditional LAMP techniques were combined with probes, which is a distinctive, sensitive, rapid, and high-throughput tool for the concurrent detection of H5, H7, and H9 subtypes of AIVs. The detection limit of the TLAMP assay was 205 copies per reaction for H5, 360 copies for H7, and 545 copies for H9, with no cross-reactivity with related avian viruses, showed excellent sensitivity and specificity. The developed innovative TLAMP method could be applied in conjunction with a portable multiplex fluorescence channel analyzer to develop a POC detection instrument, which substantially reduces the risk of laboratory contamination.

Adel et al. report a one-step multiplex real-time PCR assay designed to distinguish between very virulent infectious bursal disease virus (vvIBDV) and novel variant IBDV (nVarIBDV). The developed assay can effectively differentiate between vvIBDV and non-vvIBDV field samples, including nvarIBDV, which confirmed with VP2 gene using Sanger sequencing technology. There data presents a specific, sensitive, and straight forward multiplex real-time qRT-PCR method, which can be capable of detecting single and mixed infections of various IBDV genotypes directly from field samples.

Many IBDVs with different path-/geno-/serotypes have been confirmed to be co-circulating in China and some other Asian countries, and the current commercial vaccines cannot provide sufficient protection, especially against the newly emerging Chinese novel variant virus (nvIBDV). Wang et al. showed that the booster immunization with the current commercial IBDV vaccines still cannot provide complete protection against the nvIBDV. The oil emulsion vaccine (OEV) made by the inactivated nvIBDV isolates (YL160304) can induce high-level specific antibodies, ameliorate target organ damage, and significantly reduce the viral load of the challenged virus in the vaccinated chickens and provide complete protection against the nvIBDV. YL160304-OEV (A-nv/B-HLJ0504-like) can provide a broader protective spectrum against different nvIBDV strains.

Chen et al. designed specific primers and probes according to the sequence characteristics of the newly discovered duck adeno-associated virus (DAAV) and then established a TaqMan real-time PCR method (TaqMan-qPCR) for the detection of DAAV, with good sensitivity, specificity, and repeatability. These data also found a triple infection in Muscovy duck with DAAV, Muscovy duck virus (MDPV) and goose parvovirus (GPV).

Currently, RT-qPCR with spin column RNA extraction is the gold standard for HPAIV surveillance, but its long reaction time and need for specialized equipment limit its effectiveness for rapid response. By combining a magnetic bead RNP purification and concentration method with an RT-RPA/PAM-independent Cas12a assay detection system, Song et al. offer an effective and rapid diagnostic method for the detection of H5Nx viruses. These data exhibited high specificity, yielding positive results solely for H5Nx viruses among various influenza A virus subtypes.

Chen et al. developed and optimized an indirect ELISA method based on the prokaryotic-expressed recombinant capsid protein (ΔCap-iELISA) of goose circovirus (GoCV). The method demonstrated high sensitivity, specificity, and reproducibility. Further analysis indicated a significant correlation between age and the positive rate of GoCV antibodies among geese.

In conclusion, we would like to express all our gratitude to all 64 researchers who have contributed to this Research Topic by sharing their valuable resource from the “*Emerging diseases and diagnostics in poultry production*” perspective. We also extend our thanks to the reviewers and staff of Frontiers in Veterinary Science, whose efforts have ensured the successful completion of this Research Topic.
